# Crystal structure of (*Z*)-*N*′-[1-(3-methyl-5-oxo-1-phenyl-1,5-di­hydro-4*H*-pyrazol-4-yl­idene)prop­yl]benzene­sulfono­hydrazide

**DOI:** 10.1107/S2056989015007094

**Published:** 2015-04-15

**Authors:** Chuan-Chuan He, Guan-Cheng Xu

**Affiliations:** a Institute of Applied Chemistry, Xinjiang University, Urumqi, 830046 Xinjiang, People’s Republic of China

**Keywords:** crystal structure, pyrazolone derivative, hydrogen bonding, polymer chain

## Abstract

The title compound, C_19_H_20_N_4_O_3_S, was synthesized by refluxing equimolar amounts of 1-phenyl-3-methyl-4-propionylpyrazol-5-one and benzene­sulfonyl hydrazide in ethanol. The compound crystallizes in the keto form and the carbonyl O atom forms an intra­molecular N—H⋯O hydrogen bond with the neighbouring NH group. There is also C—H⋯O short contact involving the neighbouring phenyl ring. Probably as a result of this, the phenyl ring is inclined to the pyrazolone ring by only 7.58 (12)°. The dihedral angle between the phenyl ring and the benzene­sulfonyl ring is 22.78 (11)°. In the crystal, mol­ecules are linked by pairs of N—H⋯O hydrogen bonds, forming inversion dimers with an *R* ^2^_2_(14) ring motif. The dimers are linked *via* pairs of C—H⋯O hydrogen bonds, forming chains propagating along [100].

## Abstract 2

The title compound crystallizes in the keto form and the carbonyl O atom forms an intra­molecular N—H⋯O hydrogen bond with the neighbouring NH group. In the crystal, mol­ecules are linked by pairs of N—H⋯O hydrogen bonds to form inversion dimers, which are linked *via* pairs of C—H⋯O hydrogen bonds, forming chains propagating along [100].

## Chemical context   

Many pyrazolo­nes and their derivatives possess biological and pharmaceutical activities, such as anti­cancer, anti­tumor and anti­fungal activities as well as the inhibition of lipid peroxidation (Wang *et al.*, 1991 ▸; Yu *et al.*, 1993 ▸; Padhyé & Kauffman, 1985 ▸; Yang *et al.*, 1992 ▸). Among them, the 4-acyl pyrazolone derivatives have aroused great scientific inter­est because of their relatively simple synthesis, wide availability and structural versatility (Raman *et al.*, 2001 ▸; Yoshikuni, 1999 ▸; Uzoukwu *et al.*, 1996 ▸; Yang *et al.*, 2000 ▸).
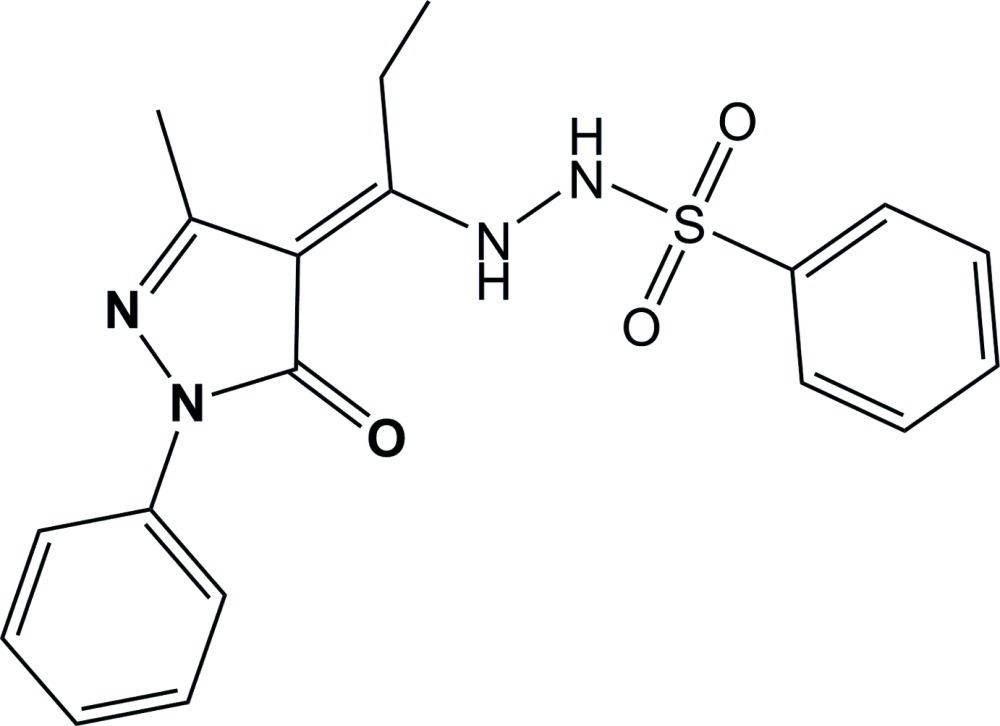


 In recent years, we have devoted our efforts to the design and synthesis of 4-acyl pyrazolone derivatives and their transition metal complexes (Zhang *et al.*, 2004 ▸; Xu *et al.*, 2013 ▸; Yi *et al.*, 2014 ▸; Li *et al.*, 2013 ▸). Such 4-acyl pyrazolone derivatives can form different types of complexes due to the multiple coordination sites and the tautomeric enol-to-keto effect. Furthermore, some of complexes have been shown to have strong anti­bacterial activity. For example, the copper complex [Cu*L*(EtOH)] [where *L* is the *N*-(1-phenyl-3-methyl-4-propenyl­idene-5-pyrazolone)salicyl­idene hydrazide anion] may be a promising drug for cancer chemotherapy (Wang *et al.*, 2007 ▸). This has encouraged us to investigate more 4-acyl pyrazolone derivatives and herein we report on the synthesis and crystal structure of the title compound.

## Structural commentary   

**Figure 1:**
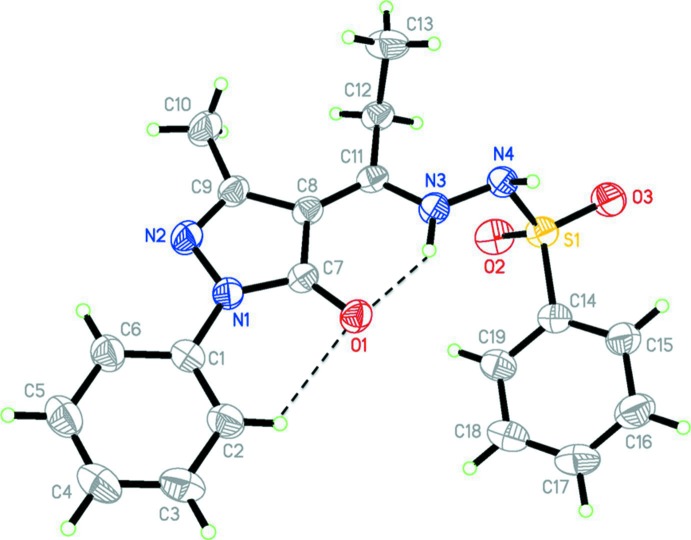
The mol­ecular structure of the title compound, with the atom labelling. Displacement ellipsoids are drawn at the 30% probability level.

The mol­ecular structure of the title compound is shown in Fig. 1 ▸. The bond lengths and angles are close to the expected values. For example, the C7—O1 bond length of 1.259 (2) Å is in good agreement with that for a C=O double bond. The C9—N2 bond length of 1.298 (3) Å is consistent with that for a normal C=N double bond, which indicates that the compound exists in the keto form. In addition, the C11—N3 bond length of 1.335 (2) Å, is very close to that for a C—N single bond. The C8—C11 bond [1.387 (3) Å] approaches the normal C=C bond length. These results indicate that the compound does not adopt the structure of a Schiff base.

The carbonyl O atom, O1, forms an intra­molecular N—H⋯O hydrogen bond with the neighbouring NH group (N3—H3*A*), and there is a short intra­molecular C—H⋯O contact involving the neighbouring phenyl ring (C1–C6) (Table 1 ▸ and Fig. 1 ▸). This phenyl ring is inclined to the pyrazolone ring (N1/N2/C7–C9) by 7.58 (12)°, which is close to the value of 6.2 (2)° reported for a related compound, 4-iso­propyl­idene-3-methyl-1-(3-nitro­phen­yl)-1*H*-pyrazol-5(4*H*)-one, which also exists in the keto form (Wardell *et al.*, 2007 ▸). The dihedral angle between the phenyl ring and the benzene­sulfonyl ring (C14–C19) is 22.78 (11)°. Hence, the whole mol­ecule is non-planar, with the torsion angle about the hydrazide bond, C11—N3—N4—S1, being −105.91 (18)°.

## Supra­molecular features   

**Figure 2:**
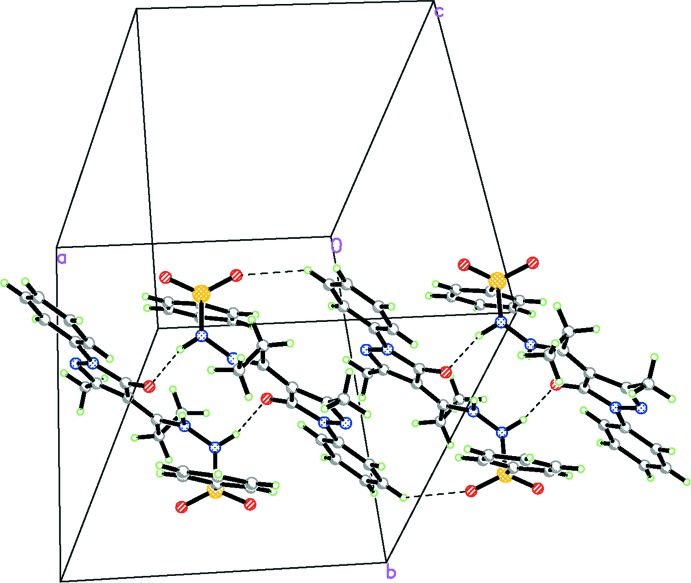
A view of the crystal packing of the title compound, with the hydrogen bonds shown as dashed lines (see Table 1 ▸ for details).

In the crystal, atom N4 acts as a donor and forms an N—H⋯O hydrogen bond with atom O1^i^ (Table 1 ▸). Mol­ecules are linked by pairs of these hydrogen bonds, forming inversion dimers with an 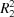
(14) ring motif. Neighbouring dimers are linked by pairs of C—H⋯O hydrogen bonds, forming chains propagating along [100] (Table 1 ▸ and Fig. 2 ▸).

## Synthesis and crystallization   

1-Phenyl-3-methyl-4-propionyl-pyrazolone-5 (20 mmol, 4.6 g) was dissolved in 25 mL of hot anhydrous ethanol, and an ethanol solution of benzene­sulfonyl hydrazide (20 mmol, 3.4 g) was slowly added with constant stirring. After adding a few drops of glacial acetic acid as catalyst, the mixture was refluxed for 4 h. After cooling, the precipitate that had formed was collected by filtration. A light-yellow product was obtained (yield 87%; m.p.: 483–484 K). Yellow block-like crystals, suitable for X-ray diffraction analysis, were obtained from a methanol solution upon slow evaporation at room temperature.

## Refinement   

Crystal data, data collection and structure refinement details are summarized in Table 2 ▸. The NH H atoms were located in a difference Fourier map and refined as riding atoms. C-bound H atoms were positioned geometrically and refined as riding: C—H = 0.93–0.97 Å with *U*_iso_(H) = 1.5*U*_eq_(C) for methyl H atoms and 1.2*U*_eq_(N,C) for other H atoms.

## Supplementary Material

Crystal structure: contains datablock(s) I, Global. DOI: 10.1107/S2056989015007094/su5112sup1.cif

Structure factors: contains datablock(s) I. DOI: 10.1107/S2056989015007094/su5112Isup2.hkl

Supporting information file. DOI: 10.1107/S2056989015007094/su5112Isup3.cml

CCDC reference: 1056718

Additional supporting information:  crystallographic information; 3D view; checkCIF report

## Figures and Tables

**Table 1:** Hydrogen-bond geometry (, )

*D*H*A*	*D*H	H*A*	*D* *A*	*D*H*A*
N3H3*A*O1	0.92	1.88	2.667(2)	142
C2H2O1	0.93	2.35	2.958(3)	123
N4H4*A*O1^i^	0.93	1.90	2.800(2)	162
C5H5O2^ii^	0.93	2.56	3.299(3)	137

Symmetry codes: (i) 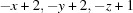; (ii) 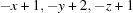.

**Table 2:** Experimental details

Crystal data	
Chemical formula	C_19_H_20_N_4_O_3_S
*M*_r_	384.45
Crystal system, space group	Monoclinic, *P*2_1_/*n*
Temperature (K)	295
*a*, *b*, *c* ()	10.601(2), 16.954(3), 11.246(2)
()	107.19(3)
*V* (^3^)	1931.0(7)
*Z*	4
Radiation type	Mo *K*
(mm^1^)	0.19
Crystal size (mm)	0.22 0.21 0.20

Data collection	
Diffractometer	Rigaku R-AXIS SPIDER
Absorption correction	Multi-scan (*ABSCOR*; Higashi, 1995 ▸)
*T*_min_, *T*_max_	0.959, 0.962
No. of measured, independent and observed [*I* > 2(*I*)] reflections	14175, 3339, 2574
*R*_int_	0.024
(sin /)_max_ (^1^)	0.595

Refinement	
*R*[*F* ^2^ > 2(*F* ^2^)], *wR*(*F* ^2^), *S*	0.040, 0.131, 1.08
No. of reflections	3339
No. of parameters	247
No. of restraints	6
H-atom treatment	H-atom parameters constrained
_max_, _min_ (e ^3^)	0.19, 0.26

Computer programs: *RAPID-AUTO* (Rigaku, 2004 ▸), *SHELXS97*, *SHELXL97* and *SHELXTL* (Sheldrick, 2008 ▸) and *PLATON* (Spek, 2009 ▸).

## supplementary crystallographic information

### Crystal data

**Table:**

C_19_H_20_N_4_O_3_S	*F*(000) = 808
*M**_r_* = 384.45	*D*_x_ = 1.322 Mg m^−^^3^
Monoclinic, *P*2_1_/*n*	Melting point: 483 K
Hall symbol: -P 2yn	Mo *K*α radiation, λ = 0.71073 Å
*a* = 10.601 (2) Å	Cell parameters from 11125 reflections
*b* = 16.954 (3) Å	θ = 3.1–27.5°
*c* = 11.246 (2) Å	µ = 0.19 mm^−^^1^
β = 107.19 (3)°	*T* = 295 K
*V* = 1931.0 (7) Å^3^	Block, yellow
*Z* = 4	0.22 × 0.21 × 0.20 mm

### Data collection

**Table:**

Rigaku R-AXIS SPIDER diffractometer	3339 independent reflections
Radiation source: fine-focus sealed tube	2574 reflections with *I* > 2σ(*I*)
Graphite monochromator	*R*_int_ = 0.024
ω oscillation scans	θ_max_ = 25.0°, θ_min_ = 3.1°
Absorption correction: multi-scan (*ABSCOR*; Higashi, 1995)	*h* = −12→12
*T*_min_ = 0.959, *T*_max_ = 0.962	*k* = −20→20
14175 measured reflections	*l* = −13→12

### Refinement

**Table:**

Refinement on *F*^2^	Secondary atom site location: difference Fourier map
Least-squares matrix: full	Hydrogen site location: inferred from neighbouring sites
*R*[*F*^2^ > 2σ(*F*^2^)] = 0.040	H-atom parameters constrained
*wR*(*F*^2^) = 0.131	*w* = 1/[σ^2^(*F*_o_^2^) + (0.0775*P*)^2^ + 0.2317*P*] where *P* = (*F*_o_^2^ + 2*F*_c_^2^)/3
*S* = 1.08	(Δ/σ)_max_ = 0.002
3339 reflections	Δρ_max_ = 0.19 e Å^−^^3^
247 parameters	Δρ_min_ = −0.26 e Å^−^^3^
6 restraints	Extinction correction: *SHELXL97* (Sheldrick, 2008), Fc^*^=kFc[1+0.001xFc^2^λ^3^/sin(2θ)]^-1/4^
Primary atom site location: structure-invariant direct methods	Extinction coefficient: 0.016 (2)

### Special details

**Table:**

Experimental. Jacobson, *R*. (1998) Private communication
Geometry. All e.s.d.'s (except the e.s.d. in the dihedral angle between two l.s. planes) are estimated using the full covariance matrix. The cell e.s.d.'s are taken into account individually in the estimation of e.s.d.'s in distances, angles and torsion angles; correlations between e.s.d.'s in cell parameters are only used when they are defined by crystal symmetry. An approximate (isotropic) treatment of cell e.s.d.'s is used for estimating e.s.d.'s involving l.s. planes.
Refinement. Refinement of *F*^2^ against ALL reflections. The weighted *R*-factor *wR* and goodness of fit *S* are based on *F*^2^, conventional *R*-factors *R* are based on *F*, with *F* set to zero for negative *F*^2^. The threshold expression of *F*^2^ > σ(*F*^2^) is used only for calculating *R*-factors(gt) *etc*. and is not relevant to the choice of reflections for refinement. *R*-factors based on *F*^2^ are statistically about twice as large as those based on *F*, and *R*- factors based on ALL data will be even larger.

### Fractional atomic coordinates and isotropic or equivalent isotropic displacement parameters (Å^2^)

**Table:**

	*x*	*y*	*z*	*U*_iso_*/*U*_eq_	
C1	0.51056 (18)	0.94366 (12)	0.32605 (18)	0.0557 (5)	
N1	0.60151 (15)	0.92936 (10)	0.44496 (15)	0.0567 (4)	
O1	0.78565 (13)	1.00662 (8)	0.44919 (12)	0.0578 (4)	
S1	1.10584 (5)	1.10154 (3)	0.79571 (4)	0.0588 (2)	
C2	0.5311 (2)	1.00300 (15)	0.2494 (2)	0.0724 (6)	
H2	0.6053	1.0352	0.2752	0.087*	
N2	0.56682 (17)	0.87417 (11)	0.52278 (17)	0.0663 (5)	
O2	1.00939 (16)	1.10824 (10)	0.85989 (14)	0.0776 (5)	
C3	0.4398 (3)	1.01385 (17)	0.1336 (2)	0.0815 (7)	
H3	0.4538	1.0532	0.0813	0.098*	
N3	0.97144 (15)	0.98778 (9)	0.66664 (14)	0.0547 (4)	
H3A	0.9375	1.0074	0.5869	0.066*	
O3	1.24221 (15)	1.11194 (9)	0.85898 (14)	0.0796 (5)	
C4	0.3288 (2)	0.96752 (17)	0.0949 (2)	0.0838 (8)	
H4	0.2683	0.9752	0.0170	0.101*	
N4	1.09686 (15)	1.00946 (9)	0.74215 (14)	0.0535 (4)	
H4A	1.1524	1.0060	0.6915	0.064*	
C5	0.3086 (2)	0.91035 (17)	0.1718 (2)	0.0815 (7)	
H5	0.2329	0.8794	0.1464	0.098*	
C6	0.3989 (2)	0.89727 (13)	0.2873 (2)	0.0676 (6)	
H6	0.3843	0.8574	0.3385	0.081*	
C7	0.72508 (18)	0.95903 (11)	0.49872 (17)	0.0501 (4)	
C8	0.76992 (18)	0.92317 (11)	0.61970 (17)	0.0503 (4)	
C9	0.66454 (19)	0.87097 (12)	0.62447 (19)	0.0589 (5)	
C10	0.6554 (2)	0.81416 (16)	0.7229 (2)	0.0835 (7)	
H10A	0.5741	0.7853	0.6947	0.125*	
H10B	0.6578	0.8425	0.7974	0.125*	
H10C	0.7284	0.7781	0.7399	0.125*	
C11	0.89157 (18)	0.93953 (11)	0.70462 (16)	0.0493 (4)	
C12	0.9392 (2)	0.90616 (12)	0.83395 (18)	0.0600 (5)	
H12A	0.8637	0.8913	0.8610	0.072*	
H12B	0.9879	0.9464	0.8903	0.072*	
C13	1.0270 (3)	0.83473 (15)	0.8404 (2)	0.0869 (8)	
H13A	0.9812	0.7962	0.7806	0.130*	
H13B	1.0492	0.8124	0.9224	0.130*	
H13C	1.1064	0.8504	0.8222	0.130*	
C14	1.06098 (19)	1.16434 (11)	0.66519 (18)	0.0568 (5)	
C15	1.1583 (2)	1.20180 (14)	0.6279 (2)	0.0734 (6)	
H15	1.2470	1.1931	0.6698	0.088*	
C16	1.1223 (3)	1.25235 (15)	0.5273 (2)	0.0871 (7)	
H16	1.1871	1.2779	0.5012	0.104*	
C17	0.9924 (3)	1.26498 (15)	0.4660 (2)	0.0838 (7)	
H17	0.9691	1.3005	0.4001	0.101*	
C18	0.8952 (3)	1.22547 (15)	0.5008 (2)	0.0790 (6)	
H18	0.8068	1.2326	0.4564	0.095*	
C19	0.9296 (2)	1.17543 (13)	0.60173 (19)	0.0655 (6)	
H19	0.8646	1.1493	0.6268	0.079*	

### Atomic displacement parameters (Å^2^)

**Table:**

	*U*^11^	*U*^22^	*U*^33^	*U*^12^	*U*^13^	*U*^23^
C1	0.0531 (11)	0.0625 (12)	0.0501 (11)	0.0071 (8)	0.0130 (8)	−0.0009 (9)
N1	0.0555 (9)	0.0618 (10)	0.0502 (9)	−0.0073 (7)	0.0115 (7)	0.0068 (8)
O1	0.0642 (8)	0.0615 (8)	0.0462 (7)	−0.0117 (6)	0.0141 (6)	0.0059 (6)
S1	0.0705 (4)	0.0586 (4)	0.0406 (3)	−0.0030 (2)	0.0062 (2)	−0.0046 (2)
C2	0.0652 (13)	0.0841 (16)	0.0624 (14)	0.0019 (11)	0.0100 (10)	0.0130 (12)
N2	0.0668 (11)	0.0688 (12)	0.0632 (11)	−0.0128 (8)	0.0187 (9)	0.0121 (9)
O2	0.1026 (11)	0.0837 (11)	0.0520 (9)	0.0062 (8)	0.0312 (8)	−0.0069 (8)
C3	0.0821 (17)	0.0970 (19)	0.0604 (14)	0.0190 (13)	0.0132 (12)	0.0191 (13)
N3	0.0611 (10)	0.0595 (10)	0.0388 (8)	−0.0095 (7)	0.0075 (7)	0.0013 (7)
O3	0.0780 (8)	0.0758 (10)	0.0623 (9)	−0.0147 (7)	−0.0146 (7)	−0.0001 (7)
C4	0.0763 (16)	0.100 (2)	0.0614 (15)	0.0200 (14)	−0.0012 (12)	−0.0071 (14)
N4	0.0558 (9)	0.0574 (10)	0.0437 (9)	−0.0033 (7)	0.0091 (7)	−0.0008 (7)
C5	0.0689 (15)	0.0896 (18)	0.0735 (16)	0.0018 (12)	0.0019 (12)	−0.0150 (14)
C6	0.0627 (13)	0.0680 (14)	0.0670 (14)	−0.0012 (10)	0.0112 (10)	−0.0069 (11)
C7	0.0576 (11)	0.0464 (10)	0.0469 (11)	−0.0025 (8)	0.0162 (8)	−0.0002 (8)
C8	0.0609 (11)	0.0474 (10)	0.0427 (10)	−0.0016 (8)	0.0156 (8)	0.0021 (8)
C9	0.0655 (12)	0.0557 (12)	0.0559 (12)	−0.0068 (9)	0.0185 (10)	0.0068 (9)
C10	0.0920 (17)	0.0807 (16)	0.0752 (16)	−0.0210 (12)	0.0207 (13)	0.0245 (13)
C11	0.0635 (11)	0.0435 (10)	0.0409 (10)	0.0012 (8)	0.0154 (8)	0.0002 (8)
C12	0.0749 (13)	0.0577 (12)	0.0436 (11)	−0.0016 (9)	0.0116 (9)	0.0051 (9)
C13	0.1057 (19)	0.0747 (17)	0.0699 (16)	0.0203 (13)	0.0099 (13)	0.0182 (12)
C14	0.0694 (13)	0.0503 (11)	0.0460 (11)	0.0037 (9)	0.0099 (9)	−0.0067 (8)
C15	0.0750 (14)	0.0680 (14)	0.0705 (14)	−0.0041 (11)	0.0113 (11)	0.0089 (12)
C16	0.1027 (19)	0.0792 (17)	0.0794 (17)	−0.0031 (14)	0.0272 (15)	0.0186 (14)
C17	0.1109 (19)	0.0750 (16)	0.0588 (14)	0.0157 (12)	0.0145 (13)	0.0111 (12)
C18	0.0837 (15)	0.0764 (16)	0.0664 (14)	0.0237 (11)	0.0060 (11)	0.0025 (12)
C19	0.0697 (13)	0.0644 (13)	0.0600 (13)	0.0122 (10)	0.0155 (10)	−0.0039 (10)

### Geometric parameters (Å, º)

**Table:**

C1—C6	1.380 (3)	C8—C11	1.387 (3)
C1—C2	1.383 (3)	C8—C9	1.439 (3)
C1—N1	1.419 (2)	C9—C10	1.492 (3)
N1—C7	1.366 (2)	C10—H10A	0.9600
N1—N2	1.402 (2)	C10—H10B	0.9600
O1—C7	1.259 (2)	C10—H10C	0.9600
S1—O2	1.4197 (17)	C11—C12	1.502 (3)
S1—O3	1.4211 (16)	C12—C13	1.517 (3)
S1—N4	1.6658 (17)	C12—H12A	0.9700
S1—C14	1.761 (2)	C12—H12B	0.9700
C2—C3	1.386 (3)	C13—H13A	0.9600
C2—H2	0.9300	C13—H13B	0.9600
N2—C9	1.298 (3)	C13—H13C	0.9600
C3—C4	1.374 (4)	C14—C19	1.378 (3)
C3—H3	0.9300	C14—C15	1.379 (3)
N3—C11	1.335 (2)	C15—C16	1.380 (3)
N3—N4	1.399 (2)	C15—H15	0.9300
N3—H3A	0.9241	C16—C17	1.362 (3)
C4—C5	1.357 (4)	C16—H16	0.9300
C4—H4	0.9300	C17—C18	1.380 (4)
N4—H4A	0.9335	C17—H17	0.9300
C5—C6	1.385 (3)	C18—C19	1.377 (3)
C5—H5	0.9300	C18—H18	0.9300
C6—H6	0.9300	C19—H19	0.9300
C7—C8	1.437 (3)		
			
C6—C1—C2	119.73 (19)	N2—C9—C10	118.08 (18)
C6—C1—N1	118.73 (19)	C8—C9—C10	130.27 (18)
C2—C1—N1	121.54 (18)	C9—C10—H10A	109.5
C7—N1—N2	111.31 (15)	C9—C10—H10B	109.5
C7—N1—C1	130.51 (17)	H10A—C10—H10B	109.5
N2—N1—C1	118.13 (15)	C9—C10—H10C	109.5
O2—S1—O3	120.96 (10)	H10A—C10—H10C	109.5
O2—S1—N4	106.62 (9)	H10B—C10—H10C	109.5
O3—S1—N4	103.79 (9)	N3—C11—C8	116.80 (16)
O2—S1—C14	108.53 (10)	N3—C11—C12	118.51 (16)
O3—S1—C14	109.05 (10)	C8—C11—C12	124.69 (17)
N4—S1—C14	107.02 (8)	C11—C12—C13	111.87 (18)
C1—C2—C3	119.1 (2)	C11—C12—H12A	109.2
C1—C2—H2	120.4	C13—C12—H12A	109.2
C3—C2—H2	120.4	C11—C12—H12B	109.2
C9—N2—N1	106.83 (16)	C13—C12—H12B	109.2
C4—C3—C2	121.1 (3)	H12A—C12—H12B	107.9
C4—C3—H3	119.4	C12—C13—H13A	109.5
C2—C3—H3	119.4	C12—C13—H13B	109.5
C11—N3—N4	123.07 (15)	H13A—C13—H13B	109.5
C11—N3—H3A	115.4	C12—C13—H13C	109.5
N4—N3—H3A	121.5	H13A—C13—H13C	109.5
C5—C4—C3	119.2 (2)	H13B—C13—H13C	109.5
C5—C4—H4	120.4	C19—C14—C15	120.9 (2)
C3—C4—H4	120.4	C19—C14—S1	119.79 (17)
N3—N4—S1	114.10 (12)	C15—C14—S1	119.35 (16)
N3—N4—H4A	105.7	C14—C15—C16	119.0 (2)
S1—N4—H4A	107.5	C14—C15—H15	120.5
C4—C5—C6	121.1 (2)	C16—C15—H15	120.5
C4—C5—H5	119.5	C17—C16—C15	120.4 (2)
C6—C5—H5	119.5	C17—C16—H16	119.8
C1—C6—C5	119.7 (2)	C15—C16—H16	119.8
C1—C6—H6	120.2	C16—C17—C18	120.5 (2)
C5—C6—H6	120.2	C16—C17—H17	119.7
O1—C7—N1	126.01 (17)	C18—C17—H17	119.7
O1—C7—C8	128.38 (17)	C19—C18—C17	119.7 (2)
N1—C7—C8	105.59 (16)	C19—C18—H18	120.2
C11—C8—C7	123.18 (17)	C17—C18—H18	120.2
C11—C8—C9	132.19 (17)	C18—C19—C14	119.5 (2)
C7—C8—C9	104.62 (16)	C18—C19—H19	120.3
N2—C9—C8	111.59 (18)	C14—C19—H19	120.3

### Hydrogen-bond geometry (Å, º)

**Table:**

*D*—H···*A*	*D*—H	H···*A*	*D*···*A*	*D*—H···*A*
N3—H3*A*···O1	0.92	1.88	2.667 (2)	142
C2—H2···O1	0.93	2.35	2.958 (3)	123
N4—H4*A*···O1^i^	0.93	1.90	2.800 (2)	162
C5—H5···O2^ii^	0.93	2.56	3.299 (3)	137

Symmetry codes: (i) −*x*+2, −*y*+2, −*z*+1; (ii) −*x*+1, −*y*+2, −*z*+1.

## References

[bb1] Higashi, T. (1995). *ABSCOR*. Rigaku Corporation, Tokyo, Japan.

[bb2] Li, H., Xu, G. C., Zhang, L., Guo, J. X. & Jia, D. Z. (2013). *Polyhedron*, **55**, 209–215.

[bb3] Padhyé, S. & Kauffman, G. B. (1985). *Coord. Chem. Rev.* **63**, 127–160.

[bb4] Raman, N., Kulandaisamy, A., Shunmugasundaram, A. & Jeyasubramanian, K. (2001). *Transition Met. Chem.* **26**, 131–135.

[bb5] Rigaku (2004). *RAPID-AUTO*. Rigaku Corporation, Tokyo, Japan.

[bb6] Sheldrick, G. M. (2008). *Acta Cryst.* A**64**, 112–122. 10.1107/S0108767307043930 18156677

[bb7] Spek, A. L. (2009). *Acta Cryst.* D**65**, 148–155. 10.1107/S090744490804362X PMC2631630 19171970

[bb8] Uzoukwu, B. A., Adiukwu, P. U., Al-Juaid, S. S., Hitchcock, P. B. & Smith, J. D. (1996). *Inorg. Chim. Acta*, **250**, 173–176.

[bb9] Wang, X. H., Jia, D. Z., Liang, Y. J., Yan, S. L., Ding, Y., Chen, L. M., Shi, Z., Zeng, M. S., Liu, G. F. & Fu, L. W. (2007). *Cancer Lett.* **249**, 256–270. 10.1016/j.canlet.2006.09.008 17055640

[bb10] Wang, L. F., Zhu, Y., Yang, Z. Y., Wu, J. G. & Wang, Q. (1991). *Polyhedron*, **10**, 2477–2461.

[bb11] Wardell, J. L., Skakle, J. M. S., Low, J. N. & Glidewell, C. (2007). *Acta Cryst.* C**63**, o462–o467. 10.1107/S0108270107029447 17675697

[bb12] Xu, G. C., Zhang, L., Zhang, Y. H., Guo, J. X., Shi, M. Q. & Jia, D. Z. (2013). *CrystEngComm*, **15**, 2873–2880.

[bb13] Yang, Z. Y., Wang, L. F., Wu, J. Q. & Li, X. Y. (1992). *Chin. J. Appl. Chem.* **9**, 31–36.

[bb14] Yang, Z. Y., Yang, R. D., Li, F. S. & Yu, K. B. (2000). *Polyhedron*, **19**, 2599–2604.

[bb15] Yi, L. J., Xu, G. C., Zhang, L. & Jia, D. Z. (2014). *Inorg. Chem. Commun.* **45**, 36–39.

[bb16] Yoshikuni, T. (1999). *J. Mol. Catal. A Chem.* **148**, 285–288.

[bb17] Yu, S. Y., Wang, S. X., Luo, Q. H., Wang, L. F., Peng, Z. & Gao, X. (1993). *Polyhedron*, **12**, 1093–1096.

[bb18] Zhang, L., Liu, L., Jia, D. Z., Xu, G. C. & Yu, K. B. (2004). *Inorg. Chem. Commun.* **7**, 1306–1310.

